# Highly potent natural fungicides identified in silico against the cereal killer fungus *Magnaporthe oryzae*

**DOI:** 10.1038/s41598-022-22217-w

**Published:** 2022-11-23

**Authors:** Md. Arif Khan, Md. Abdullah Al Mamun Khan, A. M. U. B. Mahfuz, Jannatul Maowa Sanjana, Asif Ahsan, Dipali Rani Gupta, M. Nazmul Hoque, Tofazzal Islam

**Affiliations:** 1 Department of Biotechnology and Genetic Engineering, University of Development Alternative, Dhaka, 1209 Bangladesh; 2 Department of Biotechnology and Genetic Engineering, Mawlana Bhashani Science and Technology University, Tangail, 1902 Bangladesh; 3 Department of Biotechnology, Bangladesh Agricultural University, Mymensingh, 2202 Bangladesh; 4 Institute of Biotechnology and Genetic Engineering (IBGE), Bangabandhu Sheikh Mujibur Rahman Agricultural University (BSMRAU), Gazipur, 1706 Bangladesh; 5 Department of Gynecology, Obstetrics and Reproductive Health, BSMRAU, Gazipur, 1706 Bangladesh

**Keywords:** Computational biology and bioinformatics, Drug discovery, Molecular biology

## Abstract

*Magnaporthe oryzae* is one of the most notorious fungal pathogens that causes blast disease in cereals, and results in enormous loss of grain production. Many chemical fungicides are being used to control the pathogen but none of them are fully effective in controlling blast disease. Therefore, there is a demand for the discovery of a new natural biofungicide to manage the blast disease efficiently. A large number of new natural products showed inhibitory activities against *M. oryzae *in vitro. To find out effective biofungicides, we performed in silico molecular docking analysis of some of the potent natural compounds targeting four enzymes namely, scytalone dehydratase, SDH1 (PDB ID:1STD), trihydroxynaphthalene reductase, 3HNR (PDB ID:1YBV), trehalose-6-phosphate synthase, Tps1 (PDB ID:6JBI) and isocitrate lyase, ICL1 (PDB ID:5E9G) of *M. oryzae* fungus that regulate melanin biosynthesis and/or appresorium formation. Thirty-nine natural compounds that were previously reported to inhibit the growth of *M. oryzae* were subjected to rigid and flexible molecular docking against aforementioned enzymes followed by molecular dynamic simulation. The results of virtual screening showed that out of 39, eight compounds showed good binding energy with any one of the target enzymes as compared to reference commercial fungicides, azoxystrobin and strobilurin. Among the compounds, camptothecin, GKK1032A2 and chaetoviridin-A bind with more than one target enzymes of *M. oryzae*. All of the compounds except tricyclazole showed good bioactivity score. Taken together, our results suggest that all of the eight compounds have the potential to develop new fungicides, and remarkably, camptothecin, GKK1032A2 and chaetoviridin-A could act as multi-site mode of action fungicides against the blast fungus *M. oryzae*.

## Introduction

Blast disease caused by the *Magnaporthe oryzae* (anamorph: *Pyricularia oryzae*), is one of the most destructive diseases of cereals including rice, wheat, barley, maize, finger millet etc.^1,2^. Approximately 10–30% of the global rice production is lost by the infections caused by this recalcitrant pathogen^3^. Although rice blast is prevalent in most rice-growing regions in the world, wheat blast was confined only to South American countries, including Brazil, Bolivia, Argentina, and Paraguay until 2015^4^. The disease was first time reported in an Asian country, Bangladesh, in February 2016^5^. Some newspapers in India have also reported the occurrence of this disease in the border districts of India that are close to wheat blast infected districts of Bangladesh^6^. Recently, this disease has also been reported in the Zambia, an African country, apart from South America and Asia^7^. Air-borne onidia is the mechanism of short-distance disease spread whereas, seed or grain trading is thought to be the mechanism of disease spread over a long distance^4,8^. Although the rate of devastation by the disease depends on several factors but it can cause yield loss of up to 100% in a congenial environment^2,9^. The genus of *M. oryzae* consists of several pathotypes and can cause blast disease on more than 50 Poaceae plants^10^. Based on host specificity, they are classified as *Oryza* pathotype (infecting rice), *Triticum* pathotype (infecting wheat), *Setaria* pathotype (infecting foxtail millet), *Lolium* pathotype (infecting ryegrass), and many other^11,12^. Although the isolates from different hosts are genetically distinct, cross-infection does occur to some extent^4,13^. As *M. oryzae Triticum* isolate of wheat can infect barley, maize, triticale, durum wheat and swamp rice grass in the laboratory conditions, whereas *M. oryzae Oryzae* isolates of rice can cause disease in the wheat plants^5,14,15^. However, the virulence of these pathogens during cross-infection in field conditions has not yet been established.

The control of blast disease is difficult and mainly achieved through the use of chemical fungicides^15–17^. However, extensive use of chemical fungicides has led to acquired resistance against fungicides^2,17^. Moreover, traditional breeding strategies take a long time to develop a resistant variety, and resistance often breakdown in field conditions after some years of release due to quick evolution of the pathogen^10,18^. Therefore, development of new fungicides by searching for bioactive natural compounds is a novel approach for managing the destructive fungal diseases. Various research studies have been carried out to estimate the antifungal potential of natural compounds against blast fungus in vitro*,* however, field efficacy of these compounds is still unknown^19–21^. Most of the natural compounds act directly on fungal cell components, whereas some compounds act as specific inhibitor of fungal cellular or metabolic process to inhibit the growth. Currently, researchers are focusing on identification of compounds that have specific and multiple inhibitors for fungal cellular process or pathogenicity related factors. Proteins that are responsible for the cellular process or pathogenicity of specific fungus would be the target for designing specific inhibitors that block the growth of the fungus. Like many other fungal pathogens, *M. oryzae* infects host by elaborating a specialized infection structure called appressorium. Melanin deposition in appresoria has been reported to contribute to rupture the host cell wall and establish host–pathogen interaction. Therefore, enzymes in the melanin biosynthetic pathway are valuable target for development of fungicides. Scytalone dehydratase, SDH1 (1STD), is an enzyme involved in the melanin biosynthesis in may phytopathogenic fungi including *M. oryzae*^22^. The SDH1 catalyzes the conversion of scytalone to 1, 3, 8-trihydroxynaphthalene, and vermelone to 1, 8-dihydroxynaphthalene. Whereas, trihydroxynaphthalene reductase, 3HNR (1YBV), an essential enzyme for the biosynthesis of fungal melanin, catalyzes the conversion of trihydroxynaphthalene to vermelone^23^. Subsequently, the 1, 8-dihydroxynaphthalene is polymerized into melanin. Moreover, during the morphogenesis of conidium to appressorium development, storage compounds like sugar and lipid present in the conidium are moved from conidium to appressorium as a source of energy required for generation of turgor pressure^24^. One such sugar, trehalose, is present in conidia of *M. oryzae* and is mobilized during appressorium formation. The biosynthesis of trehalose is partly regulated by trehalose-6-phosphate synthase, Tps1 (6JBI), and deletion of the *Tps1* gene in *M. oryzae* abolishes its ability to cause disease in rice^25–27^. Isocitrate lyase, ICL1 (5E9G), one of the principal enzymes of the glyoxylate cycle in the rice blast fungus *M. oryzae* helps cells to assimilate two-carbon compounds into the tricarboxylic acid cycle (TCA cycle) and channel these via gluconeogenesis to generate glucose. *Icl1* mutant cells impaired in germ tube emergence, appressorium development and cuticle penetration, and were less virulent compared to wild type strain^28^. A recent study showed that bromophenols isolated from the red alga *Odonthalia corymbifera* exhibited potent ICL inhibitory activity and blocked appressoria formation by *M. grisea.* It reduces blast disease severity in rice leaves caused by *M grisea*^29^.

Molecular docking and other computational study of inhibitors on their target enzymes or proteins of a pathogenic fungus can contribute a great help to estimate the potential of the inhibitors that could prevent the activity of an enzyme. In silico analyses of molecular docking and protein—ligand interaction of antifungal metabolites on target enzymes or proteins are also important for understanding the mechanism of antifungal action and their potential as a novel candidate fungicide against *M. oryzae*. In this study, we aimed to screen some recently reported inhibitory natural products against blast fungus *M. oryzae* for understanding their mechanisms of action and promise as candidate fungicides using in silico molecular docking studies on some enzymes involved in pre-inflectional development of the blast fungus. The specific objectives of this study were to (i) virtual screening and molecular docking simulation of 39 promising antifungal natural products on four enzymes viz. SDH1, 3HNR, TPS1 and ICL1 using PyRx 0.8; ii) assess fungicide-likeness; and iii) bioactivity of natural compounds using molecular dynamics (MD) simulation analysis of the protein–ligand complexes.

## Results

### Molecular docking simulations of the selected compounds

Molecular docking simulations were used to clarify the compounds' binding mode and obtain other information that could be utilized for further structural optimization^30,31^. Our selected compounds and two reference fungicide compounds were docked against the four different target enzymes scytalone dehydratase or SDH1 (1STD), trihydroxynaphthalene reductase (1YBV), trehalose-6-phosphate synthase 1 or Tps1 (6JBI) and isocitrate lyase enzyme or ICL1 (5E9G). The docked compounds were ranked based on the maximum occupancy of binding pocket with minimum free energy, the strength of hydrogen bonding, and other potential non-covalent interactions. Out of 39 docked molecules, top-ranking docking products were selected for further study.

**Figure 1:**
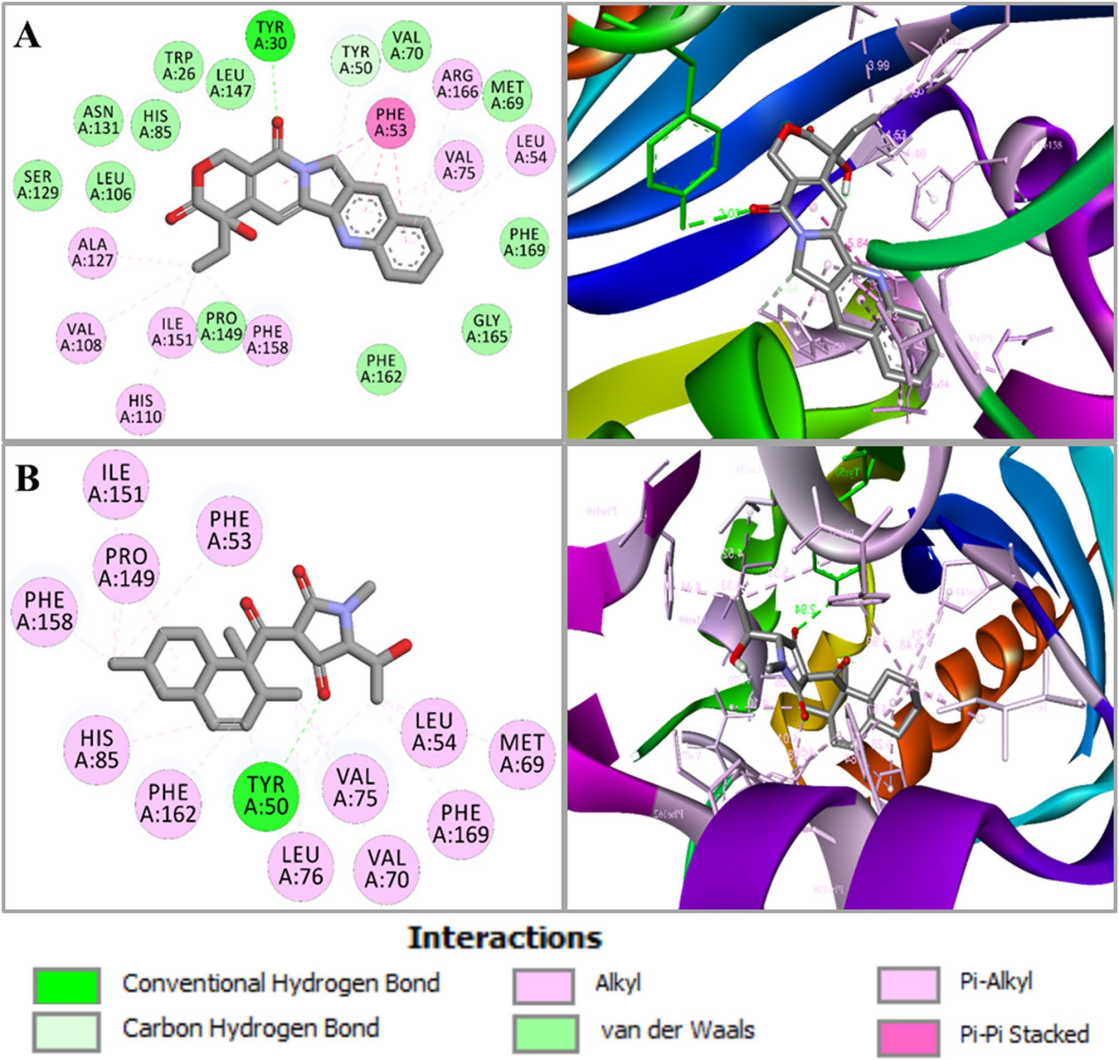
Molecular docking of the screened compounds. (**A**) Camptothecin docked in complex with scytalone dehydratase (PDB ID: 1STD); forming a hydrogen bond with side chain A:TYR30 and A:TYR50, whereas hydrophobic interactions with residues A:TYR50, A:PHE53, A:LEU54, A:VAL75, A:VAL108, A:HIS110, A:ALA127, A:ILE151, A:PHE158 and A:ARG166. (**B**) Interaction between compound cryptocin and scytalone dehydratase illustrated. A 2D interaction analysis is shown at left and 3D interaction analysis is shown at right side.

**Figure 2:**
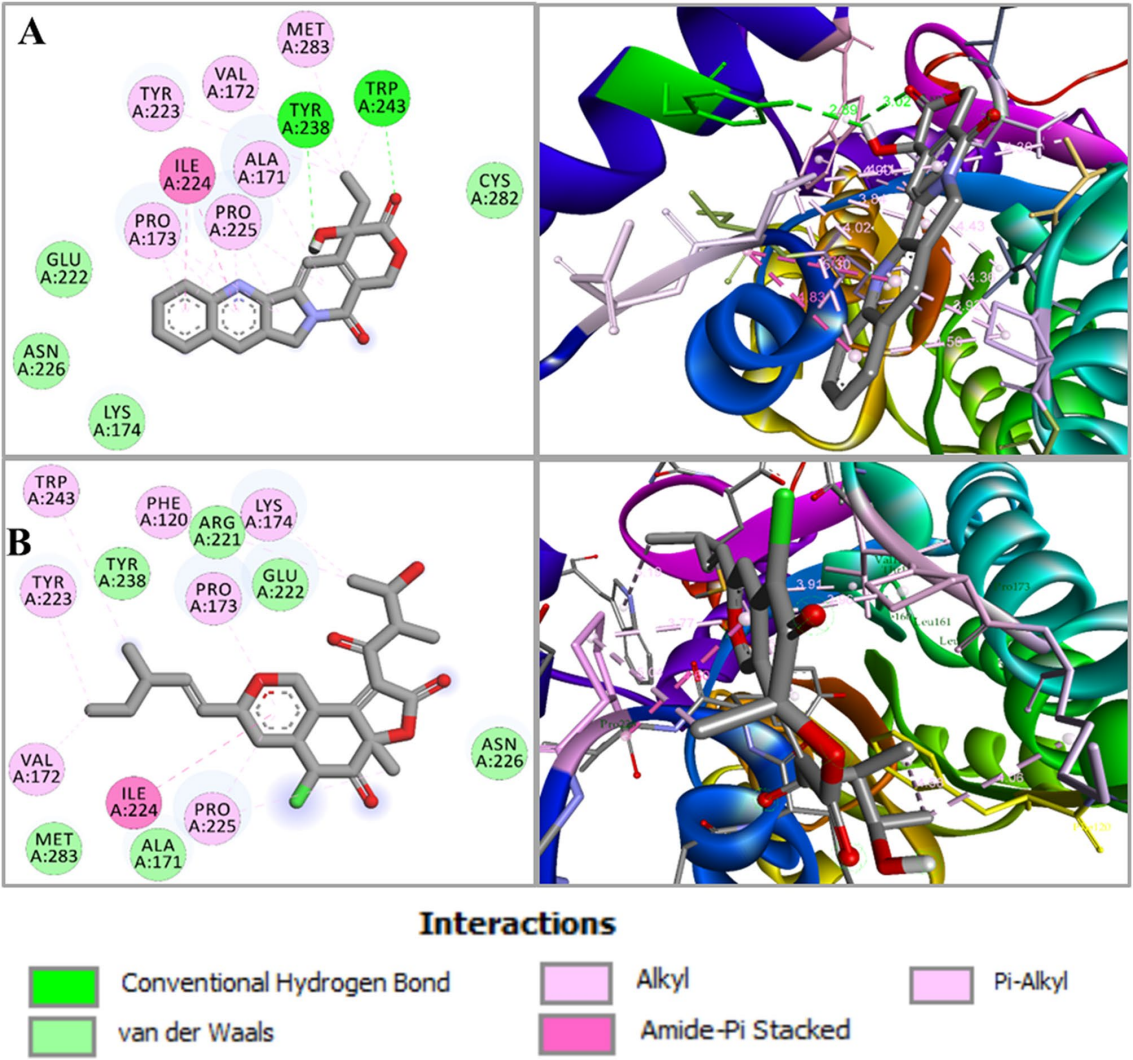
Molecular docking of the screened compounds. (**A**) Camptothecin docked in complex with trihydroxynaphthalene reductase (PDB ID: 1YBV). Camptothecin showed hydrogen bond with residues A:TRP243 and A:TYR238, and hydrophobic non bonded interactions formed with A:ILE224, A:VAL172, A:PRO225, A:TYR223, A:TRP243, A:PRO173, A:ALA171 and A:MET283. A 2D interaction analysis is shown at left and 3D interaction analysis shown at right side. (**B**) Interaction between trihydroxynaphthalene reductase and compound chaetoviridin-A illustrated.

Compounds were docked with two enzymes of the melanin pathway, scytalone dehydratase (1STD) and trihydroxynaphthalene reductase (1YBV), to inhibit the melanin pathway is responsible for appressorium formation (Table S1). Compound cryptocin, HDFO, tanzawaic-acid-L and camptothecin showed strong binding affinity − 10.1 kcal/mol, − 9.3 kcal/mol, − 9.2 kcal/mol and − 11.1 kcal/mol, respectively against scytalone dehydratase (1STD) (Table 1A). Camptothecin was bound with scytalone dehydratase (1STD) and formed a hydrogen bond with side chain A:TYR30 and A:TYR50, whereas hydrophobic interactions with residues A:TYR50, A:PHE53, A:LEU54, A:VAL75, A:VAL108, A:HIS110, A:ALA127, A:ILE151, A:PHE158, A:ARG166 (Table 1A and Fig. 1A). Rest of the compounds had interactions with amino acid residues A:SER129, A:ASN131, A:TYR50, A:VAL75, A:MET69, A:LEU76, A:ILE151, A:ALA127, A:PRO149, A:VAL70, A:LEU54, A:ARG166, A:TYR30, A:PHE53, A:PHE158, A:PHE162, A:PHE169, A:HIS85, A:VAL108, A:TRP26, A:HIS110 (Table 1A and Fig. 1B). Bond distance and type of interactions were shown in Table 2 and Table S2. All these compounds showed strong binding with scytalone dehydratase (1STD) active site residues followed by tanzawaic-acid-L, cryptocin, and HDFO, whose binding affinity was lower than camptothecin. On the other hand, compounds camptothecin, alternariol-monomethyl-ether and arohynapene-A, exhibit the highest binding affinity − 8.4 kcal/mol, − 6.4 kcal/mol and − 6.9 kcal/mol, respectively with trihydroxynaphthalene reductase (1YBV) amongst all compounds (Table 1B). Camptothecin showed hydrogen bond with residues A:TRP243 and A:TYR23 whereas hydrophobic non bonded interactions were formed with A:ILE224, A:VAL172, A:PRO225, A:TYR223, A:TRP243, A:PRO173, A:ALA171, A:MET283 (Table 1B and Fig. 2A). Other compounds showed interaction with A:TRP243, A:GLN242, A:TYR223, A:ALA171, A:TYR238 A:PRO225, A:ILE224, A:VAL172, A:LYS174, A:PHE120, and A:PRO173 (Table 1B). Interaction between trihydroxynaphthalene reductase and compound chaetoviridin-A is shown in Fig. 2B. trihydroxynaphthalene reductase has a Ser-Tyr-Lys triad in its active site that is proposed to participate in catalysis, and our selected compound formed hydrogen bond interaction with those amino acid residues (Fig. 2B).

**Table 1:** Summary of top-ranked compounds screened against scytalone dehydratase (1STD), trihydroxynaphthalene reductase (1YBV), trehalose-6-phosphate synthase 1 or Tps1 (6JBI) and isocitrate lyase enzyme (5E9G) with their respective binding energy and interacting amino acid residues.

Compounds	Binding energy (kcal/mol)	Residues involved in hydrogen bond interaction	Residues involved in hydrophobic interaction	Other
**A. 1STD**				
Cryptocin	− 10.1	**A:TYR50**	**A:PHE53**, A:LEU54, **A:MET69**, A:VAL70**, A:VAL75, A:LEU76, A:HIS85**, A:PRO149, A:ILE151, **A:PHE158, A:PHE162, A:PHE169**	
Tanzawaic-acid-L	− 9.2	**A:SER129, A:ASN131**	**A:TYR30, A:TYR50, A:PHE53**, A:LEU54, A:VAL70, **A:VAL75**, A:PRO149, **A:PHE158, A:PHE162**, A:ARG166, **A:PHE169**	
Camptothecin	− 11.1	**A:TYR30, A:TYR50**	**A:TYR50, A:PHE53,** A:LEU54, **A:VAL75**, A:VAL108, **A:HIS110,** A:ALA127, A:ILE151, **A:PHE158,** A:ARG166	
HDFO	− 9.3	**A:TYR50**	A:TRP26, **A:TYR30, A:HIS85**, A:VAL108, **A:HIS110**, A:PRO149, **A:PHE158**	
Reference Fungicide (Azoxystrobin)	− 8.3	**A:HIS85, A:ASN131**	A:LEU54, **A:MET69, A:VAL75**, A:VAL108, A:ALA127, A:ILE151, **A:PHE158, A:PHE169**	
Reference Fungicide (Strobilurin)	− 7.9	**A:TYR50**, A:LEU106, A:ALA127, **A:SER129**	**A:PHE53**, A:LEU54, A:LYS56, A:TRP58, **A:VAL75, A:HIS85**, A:VAL108, **A:HIS110**, A:PRO149, **A:PHE158, A:PHE162**, A:ARG166, **A:PHE169**	
**B. 1YBV**				
Alternariol-monomethyl-ether	− 6.4	A:TRP243, A:GLN242, **A:TYR223**, A:ALA171, A:TYR238	A:PRO225, A:PRO173, A:ALA171	
Chaetoviridin-A	− 6.9		A:ILE224, A:VAL172, A:PRO225, A:LYS174, A:PHE120, **A:TYR223**, A:TRP243, A:PRO173	
Camptothecin	− 8.4	A:TRP243, A:TYR238	A:ILE224, A:VAL172, A:PRO225, **A:TYR223**, A:TRP243, A:PRO173, A:ALA171, A:MET283	
Reference Fungicide (Azoxystrobin)	− 5.6	A:ARG133	A:VAL117, A:ALA218	
Reference Fungicide (Strobilurin)	− 6.8	A:ARG39	A:VAL219, A:VAL118, A:ALA218, A:ARG221, A:MET215	
**C. 6JBI**				
Chaetoviridin-A	− 7.3	A:ARG289, **A:LYS294**	A:ILE251, A:ARG289, **A:LEU392**	
GKK1032A2	− 10.2	A:HIS112, A:THR182, **A:LYS294, A:MET390**	**A:HIS181, A:LEU392**	
Camptothecin	− 8.7	**A:VAL287**, **A:LYS294**, **A:ASN391, A:LEU392, A:VAL393**	A:LEU371, **A:LEU392, A:VAL393**	A:GLU396
Rocaglaol	− 7.3	**A:TYR99**, A:THR182, A:ASP388	**A:TYR99, A:TRP108**, A:HIS112, **A:HIS155, A:LEU392**	**A:ARG327**
Reference Fungicide (Azoxystrobin)	− 7.4	**A:TYR99**, A:HIS112, A:TYR154, A:THR182, A:ARG289, **A:LYS294, A:ARG327**	**A:TRP108, A:LEU392**	**A:ASP153**
Reference Fungicide (Strobilurin)	− 7.7	A:HIS112, **A:HIS181**, A:HIS212, **A:GLY389, A:MET390**	A:VAL324, A:VAL366, A:LEU371, **A:LEU392, A:VAL393**	
**D. 5E9G**				
GKK1032A2	− 9.1	A:GLN449	A:TRP440, A:ILE466, A:ALA469	
Camptothecin	− 7.0		A:LEU434, A:TRP440, A:ILE466	
Reference Fungicide (Azoxystrobin)	− 7.0		A:TYR83, A:LEU434, A:ILE466	
Reference Fungicide (Strobilurin)	− 7.8	A:TYR85, A:GLN449	A:TYR83, A:LEU434, A:TRP440, A:TYR452, A:ILE453, A:ILE466	

*Active site amino acids are bolded.

**Table 2:** Type of interactions, interacting residues and bond distance of scytalone dehydratase (1STD), trihydroxynaphthalene reductase (1YBV), trehalose-6-phosphate synthase 1 or Tps1 (6JBI) and isocitrate lyase enzyme (5E9G) with their best binding energy compound.

Compounds	Interacting amino acid residues	Bond distance (Å)	Interaction category	Type of interaction
1YBV vs. Camptothecin	A:ALA171	4.30500	Hy Bond	Pi-Alkyl
	A:VAL172	4.42792	Hy Bond	Alkyl
	A:PRO173	4.36460	Hy Bond	Alkyl
	A:PRO173	3.91795	Hy Bond	Pi-Alkyl
	A:PRO173	4.56089	Hy Bond	Pi-Alkyl
	A:TYR223	5.01426	Hy Bond	Pi-Alkyl
	A:ILE224	4.77982	Hy Bond	Amide-Pi Stacked
	A:ILE224	4.82795	Hy Bond	Amide-Pi Stacked
	A:PRO225	3.83534	Hy Bond	Alkyl
	A:PRO225	5.29914	Hy Bond	Pi-Alkyl
	A:PRO225	4.40528	Hy Bond	Pi-Alkyl
	A:PRO225	4.02346	Hy Bond	Pi-Alkyl
	A:TYR238	2.88848	H Bond	Conventional H Bond
	A:TRP243	3.02142	H Bond	Conventional H Bond
	A:TRP243	4.71108	Hy Bond	Pi-Alkyl
	A:TRP243	4.90207	Hy Bond	Pi-Alkyl
	A:MET283	3.97625	Hy Bond	Alkyl
1STD vs. Camptothecin	A:TYR30	3.03114	H Bond	Conventional H Bond
	A:TYR50	2.83592	H Bond	Carbon H Bond
	A:TYR50	4.5893	Hy Bond	Pi-Alkyl
	A:PHE53	5.47049	Hy Bond	Pi-Alkyl
	A:LEU54	5.42091	Hy Bond	Pi-Alkyl
	A:VAL75	4.62589	Hy Bond	Pi-Alkyl
	A:VAL108	3.99317	Hy Bond	Alkyl
	A:HIS110	4.60518	Hy Bond	Pi-Alkyl
	A:ALA127	3.55428	Hy Bond	Alkyl
	A:ILE151	4.5329	Hy Bond	Alkyl
	A:PHE158	4.45972	Hy Bond	Pi-Alkyl
	A:ARG166	5.48424	Hy Bond	Pi-Alkyl
5E9G vs. GKK1032A2	A:TRP440	3.68425	Hy Bond	Pi-Pi Stacked
	A:GLN449	2.43557	H Bond	Conventional H Bond
	A:ILE466	5.34579	Hy Bond	Alkyl
	A:ALA469	3.86322	Hy Bond	Alkyl
6JBI vs. GKK1032A2	A:HIS112	2.85945	H Bond	Conventional H Bond
	A:HIS181	4.11942	Hy Bond	Pi-Alkyl
	A:THR182	2.98721	H Bond	Carbon H Bond
	A:LYS294	3.46775	H Bond	Pi-Donor H Bond
	A:MET390	2.68059	H Bond	Conventional H Bond
	A:LEU392	4.47259	Hy Bond	Alkyl

*H* hydrogen, *Hy* hydrophobic.

**Figure 3:**
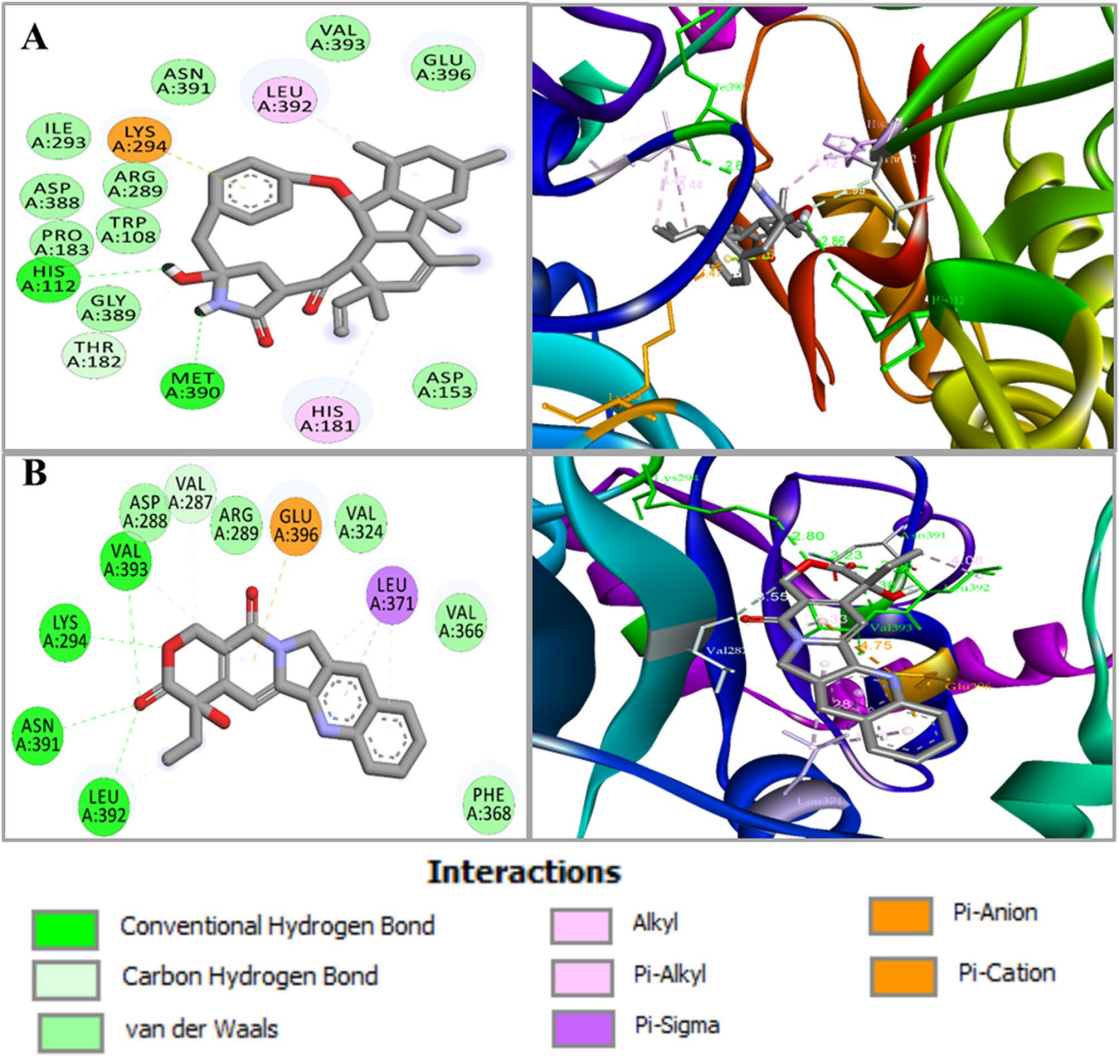
Molecular interactions of the docked complexes. (**A**) GKK1032A2 docked in complex with trehalose-6-phosphate synthase 1 or Tps1 (PDB ID: 6JBI); Hydrogen bonds favor the docking interactions of GKK1032A2 with A:HIS112, A:THR182, A:LYS294 and A:MET390, while non-bonded hydrophobic interactions are favored by A:LEU392 and A:HIS181. (**B**) Interaction between trehalose-6-phosphate synthase 1 or Tps1 and compound camptothecin illustrated. A 2D interaction analysis is shown at left and 3D interaction analysis is shown at right side.

**Figure 4:**
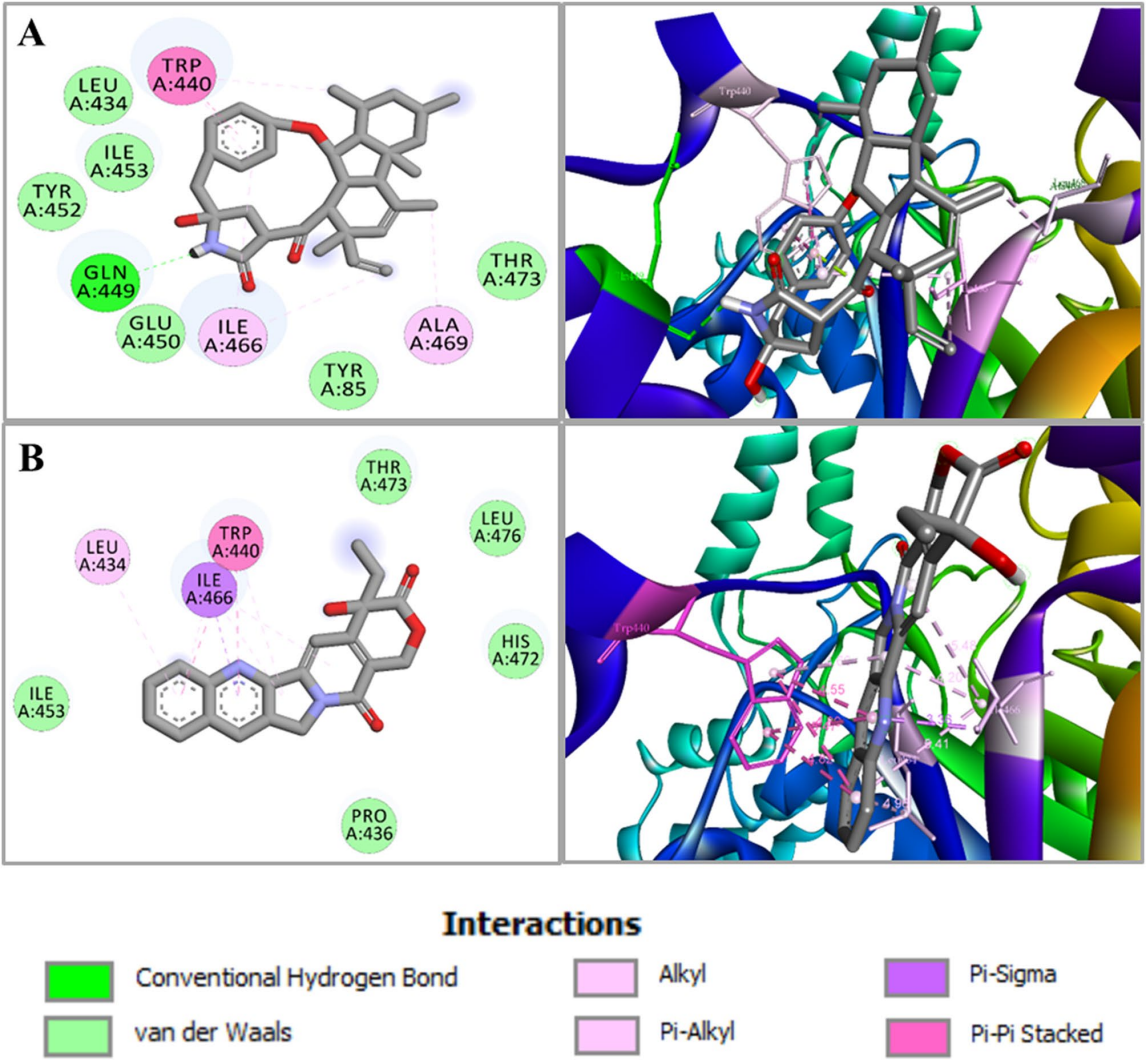
Molecular interactions of the docked complexes. (**A**) GKK1032A2 docked in complex with isocitrate lyase enzyme (PDB ID: 5E9G). The binding conformations were analyzed, and we identified that GKK1032A2 formed a hydrogen bond with A:GLN449. In addition, several residues A:TRP440, A:ILE466, A:ALA469 formed hydrophobic interactions. (**B**) Camptothecin hydrophobic interactions with residues A:LEU434, A:TRP440 and A:ILE466. A 2D interaction analysis is shown at the left and 3D interaction analysis is shown at right side.

Bond distance and type of interactions are shown in Table 2 and Table S2. Camptothecin showed the highest binding affinity and more hydrogen bonds than other compounds. Likewise, in trehalose-6-phosphate synthase 1 or Tps1 (6JBI), the compound GKK1032A2, camptothecin, chaetoviridin-A and rocaglaol have been observed to bind through meaningful bonds having binding scores of − 10.2 kcal/mol, − 8.7 kcal/mol, − 7.3 kcal/mol, and − 7.3 kcal/mol, respectively (Table 1C). Hydrogen bonds favor the docking interactions of GKK1032A2 with A:HIS112, A:THR182, A:LYS294, and A:MET390, while non-bonded hydrophobic interactions are favored by A:HIS181 and A:LEU392 (Table 1C and Fig. 3A). Other three compounds showed interactions with residues A:ARG289, A:LYS294, A:VAL287, A:ASN391, A:LEU392, A:VAL393, A:TYR99, A:THR182, A:ASP388, A:ILE251, A:LEU371, A:TRP108, A:HIS112, A:HIS155, A:GLU396, A:ARG327 and A:ASP153, as detailed in Table 1C. Interaction between trehalose-6-phosphate synthase 1 or Tps1 and compound camptothecin is illustrated in Fig. 3B. Bond distance and type of interactions are shown in Table 2 and Table S2. GKK1032A2 showed strong binding with trehalose-6-phosphate synthase 1 or Tps1 (6JBI) active site residues and highest binding affinity compared to chaetoviridin-A, camptothecin and rocaglaol. In case of the isocitrate lyase enzyme (5E9G), GKK1032A2 and camptothecin possessed the highest binding affinity − 9.1 kcal/mol and − 7.0 kcal/mol, respectively among all the compounds (Table 1D). The binding conformations were analyzed, and we identified that GKK1032A2 formed a hydrogen bond with A:GLN449. In addition, several residues such as A:TRP440, A:ILE466, A:ALA469 formed hydrophobic interactions (Table 1D and Fig. 4A). While camptothecin formed hydrophobic interactions with residues A:LEU434, A:TRP440, A:ILE466 (Table 1D and Fig. 4B).

### Fungicide likeness

Physicochemical properties of the potential compounds were analyzed to evaluate their fungicide-likeness nature. We analyzed the fungicide-likeness of the natural compounds under the well-established fundamental rule of drug-likeness (Lipinski’s rule of 5) since there is currently no such criteria for fungicides^32^. Natural compounds physicochemical properties including molecular weight, number of rotatable bonds, number of hydrogen bond acceptors, and number of hydrogen bond donors, topological polar surface area, fraction Csp3, molar refractivity, and synthetic accessibility were analyzed. The predicted results are listed in Table 3. Interestingly, all of the selected natural compounds had the molecular weight ranging from 248.32 to 434.48 (< 500) except GKK1032A2 (Table 3). The milogP values of the potential compounds were found to be below 5 (0.89 to 5.08) except compound GKK1032A2. According to Lipinski’s rule, that most “drug-like” molecules have number of hydrogen bond acceptors ≤ 10, and number of hydrogen bond donors ≤ 5. Furthermore, the number of hydrogen bond donors was < 5, and the number of hydrogen bond acceptors was < 10. Besides, TPSA of all potential compounds was observed in the range of 46.53 to 89.9 Å^2^.

**Table 3:** Physicochemical properties of selected potential compounds and reference fungicide compounds.

Compounds	MW (g/mol)	RB	HBA	HBD	miLogP	Lipinski	TPSA (Å^2^)	Synthetic accessibility
Alternariol-monomethyl-ether	272.25	1	5	2	2.74	Yes	79.9	2.83
Cryptocin	361.48	3	4	1	0.89	Yes	74.68	4.76
Chaetoviridin-A	432.89	6	6	1	3.22	Yes	89.9	5.68
GKK1032A2	503.67	1	2	4	5.08	Yes	75.63	7.28
Tanzawaic-acid-L	288.38	3	3	2	3.19	Yes	57.53	5.16
Camptothecin	348.35	1	5	1	2.03	Yes	81.2	3.84
Rocaglaol	434.48	5	6	2	4.48	Yes	77.38	4.85
HDFO	248.32	0	3	1	1.51	Yes	46.53	4.02
Azoxystrobin	403.39	8	8	0	3.38	Yes	103.56	3.42
Strobilurin	442.5	6	7	1	4.92	Yes	83.45	5.6

*MW* molecular weight, *RB* rotatable bond, *HBA* hydrogen bond acceptor, *HBD* hydrogen bond donor, *TPSA* topological surface area.

### Bioactivity score assessment of selected potential natural products

The bioactivity score of the selected compounds was predicted through the Molinspiration server (https://www.molinspiration.com/). In this prediction, biological activity measured by the bioactivity score for enzyme inhibitor was evaluated enzyme (Table 4), which are classified into three different ranges: molecule having bioactivity score > 0.00 is most likely to illustrate meaningful biological activity, while scores extending from − 0.50 to 0.00 are expected to be moderately active, and if the score is < − 0.50, it is presumed to be inactive. The bioactivity scores for the G protein-coupled receptor ligand (GPCR) were found to be most active for all of the selected compounds, except for alternariol-monomethyl-ether and GKK1032A2 that were moderately active. Moreover, the ion channel modulators' scores for tanzawaic-acid-L, HDFO, azoxystrobin, and strobilurin revealed these compounds as biologically active, and all other compounds were found to be moderately active (Table 4).

**Table 4:** Prediction of bioactivity of the selected compounds and reference fungicide compounds.

Compounds	GPCR ligand	Ion channel modulator	Kinase inhibitor	Nuclear receptor ligand	Protease inhibitor	Enzyme inhibitor
Alternariol-monomethyl-ether	− 0.41	− 0.44	− 0.25	0.23	− 0.48	0.03
Cryptocin	0.08	− 0.25	− 0.48	0.2	0.24	0.18
Chaetoviridin-A	0	− 0.35	− 0.44	0.09	0.02	0.28
GKK1032A2	− 0.16	− 0.17	− 0.76	0.13	0.07	0.05
Tanzawaic-acid-L	0.17	0.03	− 0.47	0.61	− 0.11	0.41
Camptothecin	0.46	− 0.15	0.27	0.07	− 0.1	1.11
Rocaglaol	0.18	− 0.01	− 0.1	0.4	0.01	0.1
HDFO	0.14	0.13	− 0.25	0.87	0.03	0.69
Azoxystrobin	0.25	0.03	0.09	0.25	− 0.12	0.19
Strobilurin	0.27	0	− 0.17	0.51	− 0.03	0.17

The results of kinase inhibitors score for the compounds camptothecin and azoxystrobin showed biological active scores, and other compounds were moderately active. However, the compound GKK1032A2 was found to be inactive. Moreover, the nuclear receptor score values revealed that all the compounds were biologically active according to the classification range of Linn et al.^33^. Compounds alternariol-monomethyl-ether, tanzawaic-acid-L, camptothecin, azoxystrobin, and strobilurin had moderately active score values; whereas cryptocin, chaetoviridin-A, GKK1032A2, rocaglaol, and HDFO were biologically active for protease inhibitors. The structures of all compounds had > 0.00 score values for enzyme inhibitors, and demonstrated these compounds as biologically active (Table 4).

### Molecular dynamics (MD) simulation of the protein–ligand complexes

**Figure 5:**
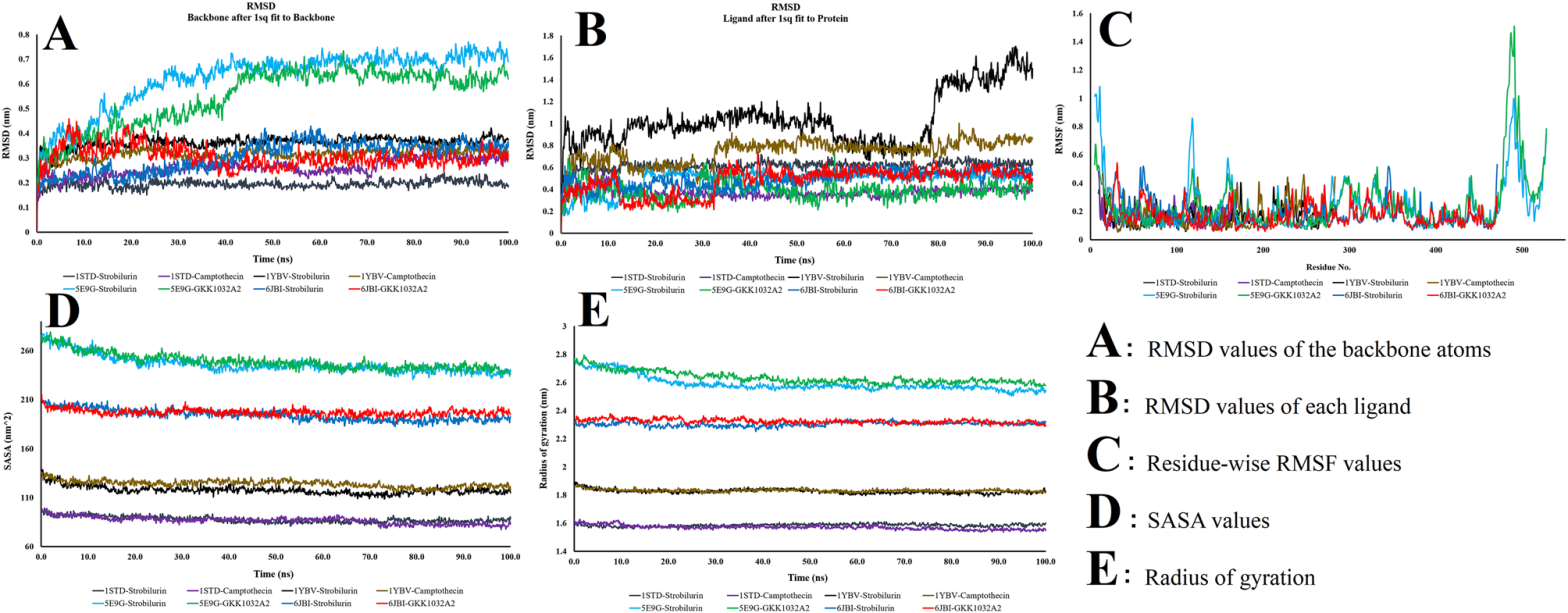
Molecular dynamics simulation of the protein-ligand complexes. (**A**) RMSD values of the backbone atoms, (**B**) RMSD values of each ligand, (**C**) residue-wise RMSF values, (**D**) SASA values, and (**E**) radius of gyration (Rg).

To elucidate the impact of the ligands on the structure, conformation, and stability of the receptors, MD simulations of the ligand–protein complexes were conducted. The RMSD, RMSF, Rg, SASA, and hydrogen bond profiles produced by the MD simulation were examined to understand more about these impacts. The stability of the selected proteins increased with decreasing fluctuations. Majority of the complexes, with the exception of 5E9G-strobirulin and 5E9G-GKK1032A2, had a constant RMSD value from the start and remained stable throughout the simulation duration. These two complexes, however, stabilized at 45 ns and continued to have an average RMSD of 0.6–1 during the remaining time (Fig. 5A). The 1YBV-camptothecin complex showed the less deviation from the reference complex (1YBV-Strobirulin). The majority of the complex maintained a constant equilibrium (RMSD remained within 0.2–0.6 nm) during the simulation, indicating strong conformational stability, based on analysis of the RMSD values of each ligand (Fig. 5B). The RMSD values of 1YBV-strobirulin complex stayed mostly stable near about 1 nm from the beginning to approximately 58 ns time. Subsequently, it showed significant deviation and RMSD value fell down to about 0.8 nm at 58 ns, which suggests a new conformation. It again changed and established a stable conformation at 80 ns and maintained it until the end of the MD simulation.

To evaluate the fluctuations in the conformations of the reference ligand–protein complex and other ligand–protein complexes, RMSF analysis was performed (Fig. 5C). RMSF values demonstrate changes in protein conformation and stability due to binding of the ligands. From the RMSF plot, we observed that most of the ligand–protein complexes showed similar profiles to the reference ligand–protein complex. The analysis of solvent accessible surface area (SASA) (Fig. 5D) showed the accessibility of the protein surface to the solvent molecules throughout the simulation. The SASA values for the reference complex and other ligand–protein complexes remained uniform during the MD simulation. The degree of compaction of a ligand–protein complex was investigated from radius of gyration (Rg). The Rg analysis (Fig. 5E) revealed that the 1STD-strobilurin and 1STD-camptothecin complexes had the lower Rg value than others. Conversely, 5E9G-strobilurin and 5E9G-GKK1032A2 showed the highest Rg value. However, all of the reference and the ligand complexes showed similar trend in case of both SASA and Rg profiles.

### Number of hydrogen bonds analysis of the protein–ligand complexes

**Figure 6:**
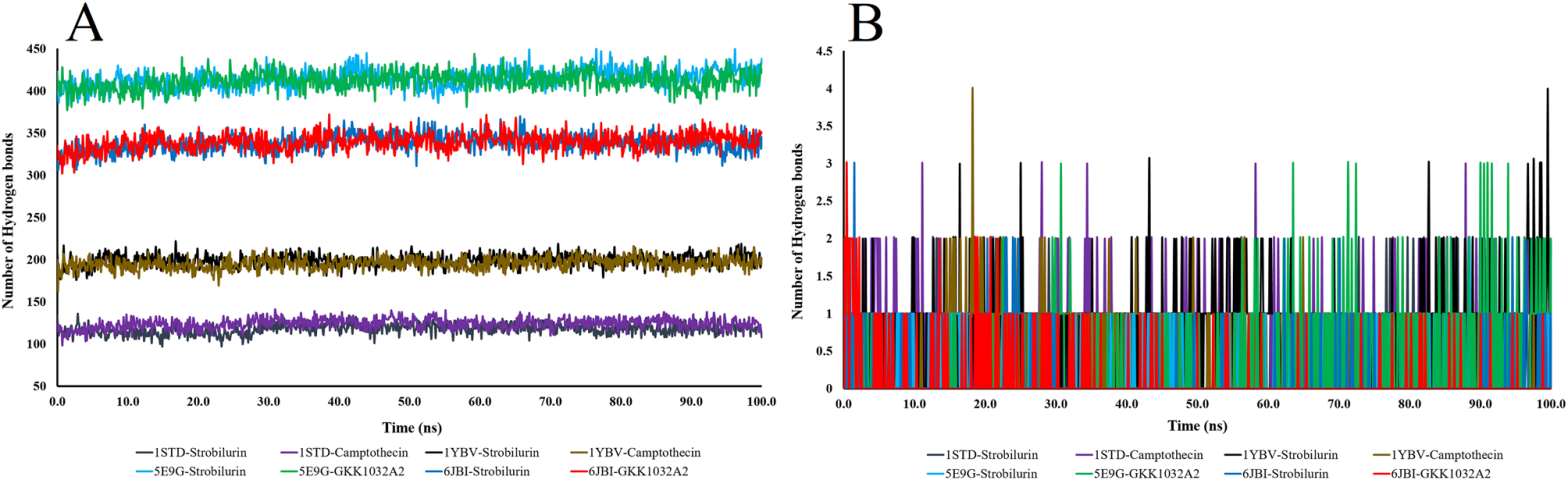
Number of hydrogen bonds within the complexes (**A**), and number of hydrogen bonds between each ligand and the receptor protein (**B**).

We further examined the number of hydrogen bonds present in the complexes (Fig. 6A). Throughout the simulation time, the protein complexes' hydrogen bond count remained constant. Additionally, the amount of hydrogen bonds that existed between each ligand and each receptor changed was also evaluated (Fig. 6B). The 6JBI-GKK1032A2 complex showed to form a maximum of four hydrogen bonds with the receptor. Therefore, GKK1032A2 was found to be bonded to the receptor just as firmly as reference strobilurin (Fig. 6B).

## Discussion

Blast caused by *M. oryzae* is a destructive disease of cereal crops that causes huge economic losses by reducing the grain yield^18^. Although the synthetic chemical fungicides provide a marginal protection against blast disease but increasing risk of health hazard and pathogen resistant is the matters of great concern. Hence, there is a need to discover eco-friendly natural fungicides alternative to the synthetic chemicals against *M. oryzae* infection that can be more effective. To find out potential fungicides against *M. oryzae*, we performed a literature-based survey, identified  39 compounds that differently inhibit the growth of *M. oryzae* fungus and then performed a comprehensive in silico study to assess the docking of these natural products on some key enzymes involved in preinfectional development of this cereal killer fungus. Protein–ligand binding affinity is essential for biological processes, as these physical and chemical interactions determine biological recognition at the molecular level. In this way, it is possible to look for a ligand capable of inhibiting or activating a specific target protein through its interaction. Therefore, it is crucial to find a ligand that binds to a target protein with high affinity^34^. The ranking criteria involved the number of hydrogen bond interactions and binding with the selected protein targets involving the binding pocket residues^32^. Based on the results of in silico study, we identified eight compounds that have high potential as new fungicide candidates. Among them, camptothecin, GKK1032A2 and chaetoviridin-A could act as multi-sites mode of action fungicides against the blast fungus *M. oryzae*. Our results for the first time demonstrated that natural products camptothecin, GKK1032A2 and chaetoviridin-A have the potential to impair preinfectional development of blast by inhibiting the key enzymes involved in melanin synthesis and/or formation of appressoria in the germinated conidia of *M. oryzae*.

One of the interesting findings of our molecular docking study is that most of the selected natural compounds bound either 1STD or 1YBV or 6JBI and/or 5E9G enzymes responsible for either melanin biosynthesis and/or appresoria formation enzymes of *M. oryzae*. Scytalone dehydratase, SDH1 (1STD) and trihydroxy naphthalene reductase, 3HNR (1YBV), two key enzymes of melanin biosynthesis pathway are found to be essential for virulence in plant pathogenic fungi^22,35^. Various studies have reported that the SDH1 and 3HNR enzymes are promising molecular target for the identification of potential inhibitors^36,37^. Several synthetic fungicides namely, carpropamid, tricyclazole, pyroquilon and phthalide have been discovered that inhibit these two enzymes of melanin biosynthesis pathway. Although these compounds were effective against *M. oryzae* but extensive uses of these fungicides lead to development of resistance. For instance, carpropamid, ((1RS,3SR)-2,2-dichloro-N-[(R)-1-(4-chlorophenyl)ethyl]-1-ethyl-3-methylcyclopropanecarboxamide), a commercial fungicide, that targets the scytalone dehydratase enzyme and it has been widely used in Japan as the chemical agent for nursery-box treatment against leaf blast of rice^16^. However, a single-point mutation in *Sdh1* in *M. oryzae* isolates causing substitution of one amino acid in the scytalone dehydratase gene showing decreased sensitivity to carpropamid^38^.

Two other enzymes namely trehalose-6-phosphate synthase, TPS1 (6JBI), and Isocitrate lyase, ICL1 (5E9G), have also been shown as good targets to design fungicides. TPS1 is the key enzyme in trehalose biosynthetic pathways that catalyzes the transfer of glucose from uridinediphospho (UDP)-glucose to glucose 6-phosphate to generate trehalose 6-phosphate (T6P). TPS1 appeared to be dispensable for development and virulence *M. oryzae*, *Fusarium verticillioides*, *Puccinia striiformis* f. sp. *tritici* and *Fusarium graminearum,* and *Tps1* mutants showed reduced pathogenicity^39,40^. Since fungal TPS1 shares minimal similarity to plant homologs, therefore, inhibition of TPS1 may serve as a promising target for the development of new strategies to control fungal diseases^41^. For example, validamycin A, a competitive inhibitor of Tps1 and has been used as a potential fungicide^42^. Isocitrate lyase (ICL), a key enzyme in carbon metabolism and is essential for the pathogenesis for both human and plant fungal pathogens^43^. It has been shown that *icl1* gene of *Leptosphaeria maculans* involved in successful host colonization of *Brassica napus*, whereas an *M. grisea Icl1* regulates virulence-associated functions such as germ tube emergence, appressorium development, and cuticle penetration. The Δ*icl* mutant exhibits less virulence than wild type and impaired virulence-associated function^44^. Several natural compounds have been identified as inhibitor of *Icl*I. Halisulfate 1, a sesterterpene sulfate, isolated from tropical sponge *Hippospongia* spp., reduces both appresorium formation and infection of rice plants by the fungus *M. grisea* by potentially binding with *Icl*1^44^. Bromophenol, another natural compound isolated from the red alga *Odonthalia corymbifera* exhibited potent ICL inhibitory activity and blocked appresoria formation of *M. grisea* in a concentration-dependent manner^29^. Joshi et al.^45^ used ICL as a molecular target to discover new antifungal compounds against *F. graminarearum* using molecular dynamic study. Four natural compounds namely, melianoninol, nimbinene, vilasinin, and fraxinellone from *Melia azedarach* identified as potent inhibitor of *ICL1*. Molecular dynamics simulation demonstrated that these four phytochemicals displayed considerable significant structural and pharmacological properties and could be probable antifungal drug candidates against *F. graminarearum*. Therefore, molecular docking and simulation studies could be utilized to predict the efficiency of binding of the ligand with biomolecules^46,47^.

In the current study, a total of 39 compounds were subjected to molecular docking study. Eight of them showed good binding affinity with the aforementioned four enzymes of *M. oryzae*. Among the eight compounds, four compounds viz. cryptocin, tanzawaic-acid-L, camptothecin, and HDFO strongly bound with 1STD whereas three natural compounds namely, alternariol-monomethyl-ether, chaetoviridin-A and camptothecin strongly bound with trihydroxy naphthalene reductase (1YBV). Chaetoviridin-A, GKK1032A2, camptothecin, and rocaglaol showed strong binding affinity for 6JBI whereas, only two compounds viz. GKK1032A2 and camptothecin were bound with 5E9G with strong affinity. Then, we have subjected all the compound for analyzing fungicide-likeness by the Lipinski’s rules of 5. Molecular weight of a chemical is an important criterion to determine its fungicides activity. The molecules that have low molecular weight (˂500) are readily transported, diffused and absorbed by the cell membrane in comparison to large molecules^48^. Molecular weight of the selected compounds was found more or less than 500 g/mol (248.32 g/mol to 506.5 g/mol). In addition, the positive Log*P* values indicate easier passage of compounds through bio-membranes and the acceptable limit is < 5^49,50^. Moreover, the lipophilic compounds easily permeable trough cell membrane by passive diffusion and bind with biomolecules to inhibit the vital metabolic enzymes in to the cell. Therefore, the membrane permeability depends on the lipophilic nature of a compound. The calculated log*P* values of natural compounds were ranged from 0.89 to 5.08, that are ideal for crossing the cell membrane. Recently, Steinberg et al.^51^ reported that C_18_-SMe_2_^+^, a mono-alkyl lipophilic cations (MALCs) having Log*P* value 2.26 easily diffuse through plasma membrane. Although molecular weight and log*P *value of some compounds were exceed the expected limit mentioned in Lipinski’s rule 5, but it would be worth mentioning that this slight increase in molecular weight and log*P* values will not have a significant impact on compound transportation and diffusion. It has been shown that the molecular mass of several FDA-approved drugs was more significant than 500 g/mol^52^. Furthermore, the number of hydrogen bond donors was less than five, and the number of hydrogen bond acceptors was less than 10^53^. Besides, TPSA of all potential compounds was observed in the range 46.53 to 89.9 Å^2^ which is also between the acceptable ranges^32^. In case of bioactivity score, all the compound poses a score value of − 0.50 to 0.00 which indicate the compounds are biologically active.

To investigate protein–ligand complexes in a physiological setting and evaluation of the conformational flexibility and stability of protein systems, MD modeling is an important tool^54,55^. For all of the ligand-receptor complexes, our MD simulations revealed stable RMSD profiles and had more binding affinity, which shed light on the structural integrity of the docked complexes. Rg profiles are used to describe protein folding and degree of compactness, and all of the complexes had tolerable variations in their matching Rg profiles^55^. Computing comparative data, including RMSD, RMSF, SASA, and Rg, were performed through analyzing the MD trajectories to calculate the change in the distance between the specified ligand/protein atoms over the whole simulation period. Interestingly, most of the protein–ligand complexes maintained strong conformational stability (RMSD remained within 0.2–0.6 nm). The presence of free energy (hydrogen bonds) between the ligand and the receptor helped to stabilize the ligand and provided a significant impact on the drug design^56^. In this study, all of systems “RMSD, RMSF, SASA, and Rg” profiles supported the rigidity and low flexibility of the studied natural products. Remarkably, one compound, GKK1032A2 showed the stronger H-bonding interactions with the active residues of the reference fungicide, strobilurin. Therefore, molecular docking and dynamics simulation of the selected ligand–protein complex and reference ligand–protein complexes predicted efficient binding^57^, and demonstrated significant potentials as new fungicide candidates.

Among the compounds, camptothecin, a very well-known alkaloid isolated from plant origin showed strong binding affinity with 1STD, 1YBV, 6JBI and 5E9G. As evident in several reports, camptothecin treatment inhibits the growth of *M. oryzae*, *Rhizoctonia solani*, *Alternaria alternata*, *Colletotrichum gloeosporioides*, *Fusarium oxysporum*, *Botrytis cinerea*, *Sphaerotheca fuliginea* and *Pseudoperonospora cubensis*^1,58^. Among the reported fungi, camptothecin was found to be most effective against *M. oryzae*, at a concentration of 1.53 μg/mL (EC50 value) whereas, it was effective against mycelial growth of *A. alternate* and *F. oxysporum* with EC50 value 250 µg/mL and for *C. gloeosporioides*, it was 500 µg/mL. The molecular simulation results showed that CPT could binds to the interface of DNA-topoisomerase I complex of *M. oryzae* and affecting the translation and carbohydrate metabolism/energy metabolism leading to cell death^58^. Four other natural compounds namely, GKK1032A2 and tanzawaic-acid-L were identified from fungus *Penicillium* sp. showed conidial germination inhibition in *M. oryzae*^59^. While the compound GKK1032A2 was effective at a concentration of 3 µg/mL, the rest of the compounds were effective at 25–50 µg/mL. However, GKK1032A2 has been found to be ineffective against other phytopathogenic fungi including *F. graminearum*, *B. cinerea* and *P. infestans* but the antifungal activity of tanzawaic-acid-L on other phytopathogenic fungi have not been tested yet. Interestingly, HDFO, (3aS,4aR,8aS,9aR)-3a-hydroxy-8a-methyl-3,5-dimethylenedecahydronaphto [2,3-b]furan-2(3H)-one, a newly identified compound from *Biscogniauxia* sp. O821 completely inhibited conidial germination of *M oryzae* at a concentration of less than 5 ppm, whereas, it significantly reduced the blast lesion formation in the presence of 5 and 10 ppm of HDFO. In addition, this compound had antifungal activity against *A. alternata*, *Cochliobolus miyabeanus*, *Colletotrichum orbiculare*, *Corynespora cassiicola*, *Fusarium oxysporum* f.sp. *conglutinans* and *Fusarium oxysporum* f.sp. *spinaciae* at > 50 ppm^60^. Like as HDFO, cryptocin also inhibit the growth of a wide range of fungi including *M. oryzae* with Minimum Inhibitory Concentration (MIC) value less than 1.0 µg/mL^20^. Engelmeier et al.^61^ reported rocaglaol inhibit germ tube formation at 1.6 and 3 µg/mL respectively. Taken together, results of our in silico study and earlier findings indicate that all the eight compounds investigated in this study could be used as lead compounds or potential biofungicides for the inhibition of aforementioned enzymes in *M. oryzae*. Validation of this predicted is needed through further in planta studies in the real field.

Although there are a number of fungicides are available for the control of many plant diseases, the blast disease caused by the different pathotypes of *M. oryzae* remain to be managed effectively. Recent studies suggest that most of the important plant pathogenic fungi acquired resistance against chemical fungicides^62,63^. Currently used fungicides generally target a single enzyme which can be overcome by single point mutation^17,38^. For instance, extensive use of strobilurin (QoI) fungicides in Brazil has led to a widespread distribution of cyt b mutations conferring resistance in strains isolated from wheat and other grasses^17^. Therefore, an alternative approach such as multi-site mode of action fungicides are needed to be discovered for the management of devastative blast disease. It is hypothesized that fungicides with multi-site mode of action would not be easily overcome by the emergence of resistance^63^. One of the significant findings of our study is that three natural products viz. camptothecin, GKK1032A2 and chaetoviridin-A have multiple enzymes target for inhibition of the blast fungus. Therefore, these compounds merit further *in-vivo* evaluation for considering them as potential fungicides or lead compounds to control blast disease.

## Conclusions

The control of blast disease in major cereal crops viz. rice, wheat, maize etc. using natural compounds is an advanced and risk-free method for disease management in agriculture. The results of present study clearly demonstrated that three natural products, camptothecin, GKK1032A2 and chaetoviridin-A are potential fungicide candidates or lead compounds for the development of effective fungicides against the most notorious blast fungus. All these compounds showed excellent binding affinities to multiple target proteins along with good numbers of H-bond and bioactivity scores compared to the currently available fungicides for blast disease management. These compounds hold ideal log*P* values and low molecular weights. Therefore, these compounds could cross the cell membranes and are able to inhibit the target enzymes in *M. oryzae* that involved in pathogenesis related factors in blast fungus. Our results convincingly suggest that at least three antifungal natural compounds viz. camptothecin, GKK1032A2 and chaetoviridin-A target multiple enzymes involved in melanin biosynthesis and/or appressoria formation in the blast fungus *M. oryzae*. Both of these processes are essential for infection of host plants by the *M. oryzae*. Further in vivo molecular and field studies are required for confirming the findings of this in silico study before recommending camptothecin, GKK1032A2 and chaetoviridin-A as fungicides or lead compounds against *M. oryzae*.

## Materials and methods

### Protein preparation

The crystal structure of *Magnaporthe oryzae* Scytalone dehydratase (PDB ID: 1STD at 2.90 Å resolution), Trihydroxynaphthalene reductase (PDB ID: 1YBV at 2.80 Å resolution), Trehalose-6-phosphate synthase 1 (Tps1) (PDB ID: 6JBI at 2.50 Å resolution) and Isocitrate lyase (PDB ID: 5E9G at 2.10 Å resolution), was retrieved from Research Collaboratory for Structural Bioinformatics (RCSB) Protein Data Bank (PDB)^64^ and considered as a template for all molecular docking simulation. A high-resolution PDB structure is better than a low-resolution structure^65^. Thus, we selected high-resolution protein compounds from the PDB for current analysis, and the physico-chemical properties of the target proteins are described in the Table S3. For protein preparation, we used Discovery Studio 2019 molecular visualization software 4.5^66^ and PyMOL 2.3.3 software^67^. First, the proteins were uploaded to the software and found unnecessary objects such as default given ligands, ions, and water molecules were removed from the PDB file. Finally, the files were saved in PDB file format for further analysis. List of the enzymes/proteins and their biological functions are given in Table S1.

### Ligand dataset preparation

**Figure 7:**
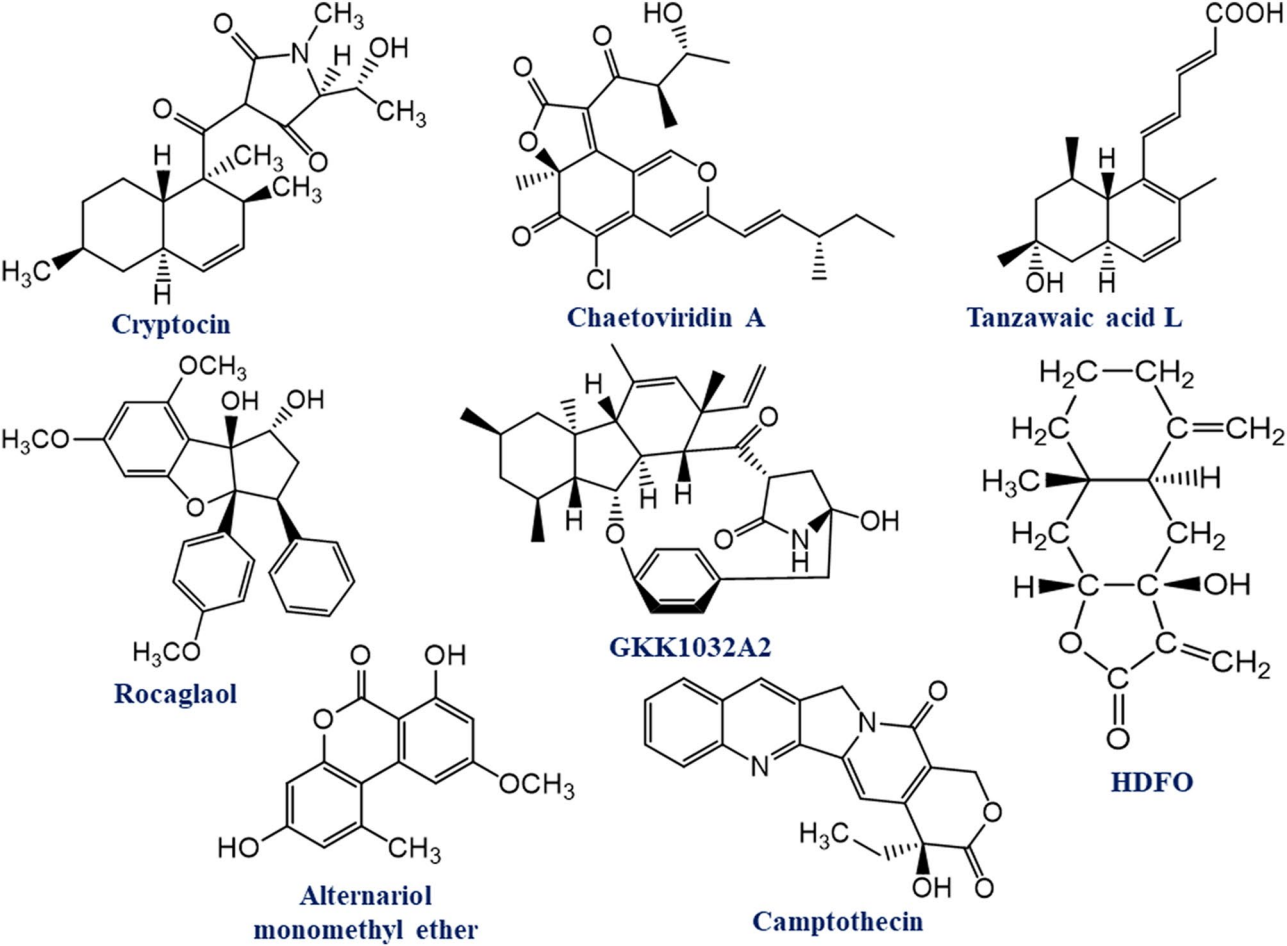
Two-dimentional (2D) chemical structure of the eight top ranked selected compounds.

The canonical smiles of 39 compounds were retrieved from the PubChem database^68^, and their 3D structures were generated using Online SMILES Translator and Structure File Generator^69^. Afterward, each of the compounds was ready as ligands for molecular docking study with the target proteins. The 2D chemical structure of best-docked compounds is illustrated in Fig. 7.

### Molecular docking simulation

In structural biology, molecular docking is a well-known and reliable technique, especially in computer-aided drug design (CADD) processes^70^. The technique ensures the best prediction of binding mode between a small molecule and a specific macromolecule^71^. It is widely established that blockade of an enzyme active site by a ligand terminates its functional activity^72^. The active site of the proteins was identified before going to the molecular docking simulation. For proteins—scytalone dehydratase^73^, trihydroxynaphthalene reductase^74^, and isocitrate lyase^75^, we determined the active sites of the amino acids from the known inhibitor of these protein. The active site of protein trehalose-6-phosphate synthase 1 was identified through metaPocket (https://projects.biotec.tu-dresden.de/metapocket/) and cross-checked by CASTp (http://sts.bioe.uic.edu/castp/index.html?2r7g) because this protein has lack of known inhibitor. In metaPocket, four methods such as LIGSITEcs, PASS, Q-SiteFinder, and SURFNET work simultaneously to rise the prediction success rate. While compared to other methods, it was observed that this consensus method can accurately predict the ligand-binding site up to 93% at the top three predictions^76^. CASTp was used to locate, delineate and measure geometric and topological properties of protein structure which included area and volume of pocket by using solvent accessible surface model (Richards’ surface) and molecular surface model (Connolly’s surface)^77^. Molecular docking simulation was carried out using PyRx 0.8 virtual screening software^78^. For simulating the best interaction, the docking was performed setting the center in axis x—31.9267, axis y—37.1866 and axis z—22.4661 with the dimension was in axis x—24.6101 Å, axis y—24.6756 Å and axis z—20.4004 Å for scytalone dehydratase (PDB ID: 1STD); center in axis x—84.7968, axis y—12.8460 and axis z—17.4490 with the dimension was in axis x—29.5738 Å, axis y—28.3518 Å and axis z—37.0650 Å for trihydroxynaphthalene reductase (PDB ID: 1YBV); center in axis x—23.3577, axis y—(− 0.9662) and axis z—25.0398 with the dimension was in axis x—61.2168 Å, axis y—98.7383 Å and axis z—95.6880 Å for Tps1 (PDB ID: 6JBI); center in axis x—(− 1.6421), axis y—34.3112 and axis z—31.0196 with the dimension was in axis x—38.5728 Å, axis y—35.1983 Å and axis z—37.2749 Å for Isocitrate lyase enzyme (PDB ID: 5E9G). We further used AutoDock 4.2.6 suit tool^72^ to validate the results of PyRx using same grid box. After docking simulation, the protein data bank partial charge and atom type (pdbqt) file format, given by Autodock as output, was saved for further protein–ligand interaction analysis.

### Protein–ligand interaction analysis

For a clear view of protein-ligand interaction of the best-docked complexes, 2D plots of protein-ligand interactions were analyzed through Discovery Studio 4.5. It generates a 2D graph of hydrogen bonds, electrostatic interactions, and hydrophobic interactions, contributing to the affinity of the drug-like molecules within the active site of *M. oryzae* proteins.

### Fungicides likeness

The physicochemical parameters of the most promising compounds were predicted using the web tool SwissADME (http://www.swissadme.ch/index.php). The predicted parameters included the number of rotatable bonds, number of hydrogen bond acceptors, number of hydrogen bond donors, partition coefficient log p (miLog P), molecular weight, synthetic accessibility, and topological polar surface area (TPSA).

### Bioactivity score prediction

The online Molinspiration Cheminformatics server (http://www.molinspiration.com) was utilized to evaluate the biological activity of selected compounds. The prediction was based on the enzyme inhibition score such as G-protein-coupled receptor (GPCR), Ion channel modulator, Kinase inhibitor, Nuclear receptor ligand, Protease inhibitor, and Enzyme inhibitor. The results are calculated according to previously published recommendations^33^. Therefore, it is recommended that if the value is equal to or greater than 0.00, the more active it will be, while if the values are between − 0.50 and 0.00, it is moderately active, and, if the score is less than − 0.50, it will be considered inactive^79,80^.

### Molecular dynamics (MD) simulation analysis

We used GROMACS^81^ and the WebGro server (https://simlab.uams.edu/) to perform molecular dynamics (MD) simulation analysis of the protein–ligand complexes. The PRODRG Server^82^ was used to create Ligand topological files employing a triclinic simulation box and the SPC water model. The system was neutralized after the addition of 0.15 M NaCl. During MD simulation, systematic analysis of the performance of the GROMOS96 43a1 force field was used. With 5000 steps of the "Steepest Descent Algorithm," energy minimization was accomplished. For equilibration in the MD simulation of biomolecules, we utilized the NVT/NPT ensemble with default parameters. Both the temperature and the pressure were set to 300 K and 1.0 bar, respectively. The simulations were run using the Leap-frog MD integrator for 100 ns. A simulation produced about 1000 frames with snapshots taken every 0.1 ns. Radius of gyration, RMSD, RMSF, SASA, and the number of Hydrogen bonds were calculated later from these snapshots^81,83^.

## Supplementary Information

Supplementary Tables.

## Data Availability

The sequence data reported in this article are available in the Protein Data Bank (https://www.rcsb.org/).
