# Supplementary material for: Highly potent natural fungicides identified in silico against the cereal killer fungus Magnaporthe oryzae
Source: Sci Rep. 2022 Nov 23;12:20232. doi: 10.1038/s41598-022-22217-w (PMC9684433; doi:10.1038/s41598-022-22217-w)
Supplement: Supplementary file 1 — Supplementary Tables. [file 41598_2022_22217_MOESM1_ESM.docx]

**Supplementary Tables**

**Table S1:** Type of interactions, interacting residues and bond distance of Scytalone dehydratase (1STD), Trihydroxynaphthalene reductase (1YBV), trehalose-6-phosphate synthase one or Tps1 (6JBI), isocitrate lyase enzyme (5E9G), with the selected fungicide compound.

| **Compounds** | **Interacting amino acid residues** | **Bond distance (Å)** | **Interaction category** | **Type of Interaction** |
| --- | --- | --- | --- | --- |
| 1STD vs. Azoxystrobin | A:HIS85 | 3.13669 | H Bond | Carbon H Bond |
|  | A:ASN131 | 2.90224 | H Bond | Conventional H Bond |
|  | A:LEU54 | 3.94836 | Hy Bond | Alkyl |
|  | A:MET69 | 4.75028 | Hy Bond | Pi-Alkyl |
|  | A:VAL75 | 3.66171 | Hy Bond | Pi-Sigma |
|  | A:VAL108 | 3.69195 | Hy Bond | Pi-Sigma |
|  | A:ALA127 | 4.86342 | Hy Bond | Pi-Alkyl |
|  | A:ILE151 | 5.01696 | Hy Bond | Pi-Alkyl |
|  | A:PHE158 | 5.18656 | Hy Bond | Pi-Pi T-shaped |
|  | A:PHE169 | 4.7673 | Hy Bond | Pi-Alkyl |
| 1STD vs. Strobilurin | A:TYR50 | 3.45555 | H Bond | Pi-Donor H Bond |
|  | A:LEU106 | 3.54787 | H Bond | Carbon H Bond |
|  | A:ALA127 | 3.18923 | H Bond | Carbon H Bond |
|  | A:SER129 | 2.61715 | H Bond | Carbon H Bond |
|  | A:PHE53 | 4.99434 | Hy Bond | Pi-Alkyl |
|  | A:LEU54 | 5.1238 | Hy Bond | Alkyl |
|  | A:LYS56 | 3.98506 | Hy Bond | Alkyl |
|  | A:TRP58 | 5.29718 | Hy Bond | Pi-Alkyl |
|  | A:VAL75 | 3.32526 | Hy Bond | Alkyl |
|  | A:HIS85 | 5.11208 | Hy Bond | Pi-Alkyl |
|  | A:VAL108 | 4.43799 | Hy Bond | Alkyl |
|  | A:HIS110 | 4.60126 | Hy Bond | Pi-Alkyl |
|  | A:PRO149 | 4.84252 | Hy Bond | Alkyl |
|  | A:PHE158 | 4.44883 | Hy Bond | Pi-Alkyl |
|  | A:PHE162 | 4.74397 | Hy Bond | Pi-Alkyl |
|  | A:ARG166 | 4.33711 | Hy Bond | Alkyl |
|  | A:PHE169 | 3.54326 | Hy Bond | Pi-Sigma |
| 1YBV vs. Azoxystrobin | A:VAL117 | 3.65329 | Hy Bond | Pi-Sigma |
|  | A:ARG133 | 3.16859 | H Bond | Conventional H Bond |
|  | A:ARG133 | 2.96348 | H Bond | Conventional H Bond |
|  | A:ALA218 | 3.63146 | Hy Bond | Pi-Alkyl |
| 1YBV vs. Strobilurin | A:ARG39 | 2.95933 | H Bond | Conventional H Bond |
|  | A:ARG39 | 3.15039 | H Bond | Conventional H Bond |
|  | A:VAL118 | 4.95132 | Hy Bond | Alkyl |
|  | A:VAL118 | 4.98844 | Hy Bond | Alkyl |
|  | A:MET215 | 4.47736 | Hy Bond | Alkyl |
|  | A:ALA218 | 3.61999 | Hy Bond | Alkyl |
|  | A:VAL219 | 3.49408 | Hy Bond | Pi-Sigma |
|  | A:ARG221 | 4.01587 | Hy Bond | Alkyl |
| 6JBI vs. Azoxystrobin | A:TYR99 | 3.48946 | H Bond | Carbon H Bond |
|  | A:TRP108 | 5.09134 | Hy Bond | Pi-Pi T-shaped |
|  | A:HIS112 | 3.68013 | H Bond | Carbon H Bond |
|  | A:ASP153 | 4.50773 | Electrostatic | Pi-Anion |
|  | A:TYR154 | 3.13047 | H Bond | Conventional H Bond |
|  | A:THR182 | 3.55883 | H Bond | Carbon H Bond |
|  | A:ARG289 | 2.83744 | H Bond | Conventional H Bond |
|  | A:LYS294 | 3.26041 | H Bond | Conventional H Bond |
|  | A:ARG327 | 3.30549 | H Bond | Conventional H Bond |
|  | A:LEU392 | 5.25434 | Hy Bond | Pi-Alkyl |
| 6JBI vs. Strobilurin | A:HIS112 | 3.54436 | H Bond | Carbon H Bond |
|  | A:HIS181 | 2.69413 | H Bond | Conventional H Bond |
|  | A:HIS212 | 2.48168 | H Bond | Conventional H Bond |
|  | A:VAL324 | 4.07098 | Hy Bond | Alkyl |
|  | A:VAL366 | 5.04402 | Hy Bond | Alkyl |
|  | A:LEU371 | 5.40251 | Hy Bond | Alkyl |
|  | A:GLY389 | 3.18744 | H Bond | Conventional H Bond |
|  | A:MET390 | 3.1601 | H Bond | Conventional H Bond |
|  | A:LEU392 | 3.81825 | Hy Bond | Alkyl |
|  | A:VAL393 | 5.31373 | Hy Bond | Pi-Alkyl |
| 5E9G vs. Azoxystrobin | A:TYR83 | 4.78837 | Hy Bond | Pi-Pi T-shaped |
|  | A:LEU434 | 4.67672 | Hy Bond | Pi-Alkyl |
|  | A:ILE466 | 3.69853 | Hy Bond | Pi-Sigma |
| 5E9G vs. Strobilurin | A:TYR83 | 5.53615 | Hy Bond | Pi-Pi T-shaped |
|  | A:TYR85 | 2.33148 | H Bond | Conventional H Bond |
|  | A:LEU434 | 3.48552 | Hy Bond | Alkyl |
|  | A:TRP440 | 3.75857 | Hy Bond | Pi-Sigma |
|  | A:GLN449 | 3.42266 | HBond | Carbon H Bond |
|  | A:TYR452 | 5.19262 | Hy Bond | Pi-Alkyl |
|  | A:ILE453 | 4.79261 | Hy Bond | Alkyl |
|  | A:ILE466 | 4.75045 | Hy Bond | Alkyl |

H= Hydrogen, Hy= Hydrophobic

**Table S2:** Type of interactions, interacting residues and bond distance of Scytalone dehydratase (1STD), Trihydroxynaphthalene reductase (1YBV), trehalose-6-phosphate synthase 1 or Tps1 (6JBI), isocitrate lyase enzyme (5E9G), with the other selected compounds.

| **Compounds** | **Interacting amino acid residues** | **Bond distance (Å)** | **Interaction category** | **Type of Interaction** |
| --- | --- | --- | --- | --- |
| 1STD vs. Tanzawaic-acid-L | A:SER129 | 3.08265 | H Bond | Conventional H Bond |
|  | A:ASN131 | 2.8417 | H Bond | Conventional H Bond |
|  | A:TYR30 | 4.59697 | Hy Bond | Pi-Alkyl |
|  | A:TYR50 | 4.73704 | Hy Bond | Pi-Alkyl |
|  | A:PHE53 | 4.62421 | Hy Bond | Pi-Alkyl |
|  | A:LEU54 | 4.17912 | Hy Bond | Alkyl |
|  | A:VAL70 | 4.8654 | Hy Bond | Alkyl |
|  | A:VAL75 | 4.64544 | Hy Bond | Alkyl |
|  | A:PRO149 | 5.46843 | Hy Bond | Alkyl |
|  | A:PHE158 | 4.65502 | Hy Bond | Pi-Alkyl |
|  | A:PHE162 | 3.52365 | Hy Bond | Pi-Alkyl |
|  | A:ARG166 | 4.52436 | Hy Bond | Alkyl |
|  | A:PHE169 | 4.96035 | Hy Bond | Pi-Alkyl |
| 1STD vs. Cryptocin | A:TYR50 | 2.94118 | H Bond | Conventional H Bond |
|  | A:PHE53 | 4.92634 | Hy Bond | Pi-Alkyl |
|  | A:LEU54 | 4.5204 | Hy Bond | Alkyl |
|  | A:MET69 | 4.32967 | Hy Bond | Alkyl |
|  | A:VAL70 | 4.55526 | Hy Bond | Alkyl |
|  | A:VAL75 | 4.10487 | Hy Bond | Alkyl |
|  | A:LEU76 | 4.0052 | Hy Bond | Alkyl |
|  | A:HIS85 | 4.84359 | Hy Bond | Pi-Alkyl |
|  | A:PRO149 | 4.21237 | Hy Bond | Alkyl |
|  | A:ILE151 | 4.74252 | Hy Bond | Alkyl |
|  | A:PHE158 | 4.67359 | Hy Bond | Pi-Alkyl |
|  | A:PHE162 | 5.08029 | Hy Bond | Pi-Alkyl |
|  | A:PHE169 | 4.40797 | Hy Bond | Pi-Alkyl |
| 1STD vs. HDFO | A:TYR50 | 2.87913 | H Bond | Conventional H Bond |
|  | A:TRP26 | 5.03721 | Hy Bond | Pi-Alkyl |
|  | A:TYR30 | 5.18836 | Hy Bond | Pi-Alkyl |
|  | A:HIS85 | 4.8075 | Hy Bond | Pi-Alkyl |
|  | A:VAL108 | 4.03815 | Hy Bond | Alkyl |
|  | A:HIS110 | 4.37553 | Hy Bond | Pi-Alkyl |
|  | A:PRO149 | 4.83225 | Hy Bond | Alkyl |
|  | A:PHE158 | 5.08614 | Hy Bond | Pi-Alkyl |
| 1YBV vs. Alternariol-monomethyl-ether | A:ALA171 | 3.67028 | H Bond | Carbon H Bond |
|  | A:ALA171 | 4.43912 | Hy Bond | Pi-Alkyl |
|  | A:PRO173 | 4.36385 | Hy Bond | Alkyl |
|  | A:TYR223 | 1.93436 | H Bond | Conventional H Bond |
|  | A:PRO225 | 4.76387 | Hy Bond | Alkyl |
|  | A:PRO225 | 4.66469 | Hy Bond | Alkyl |
|  | A:PRO225 | 4.48278 | Hy Bond | Pi-Alkyl |
|  | A:TYR238 | 3.70343 | H Bond | Pi-Donor H Bond |
|  | A:TYR238 | 3.96174 | H Bond | Pi-Donor H Bond |
|  | A:GLN242 | 2.09761 | H Bond | Conventional H Bond |
|  | A:TRP243 | 2.94298 | H Bond | Conventional H Bond |
| 1YBV vs. Chaetoviridin-A | A:PHE120 | 4.58394 | Hy Bond | Pi-Alkyl |
|  | A:VAL172 | 3.9096 | Hy Bond | Alkyl |
|  | A:PRO173 | 3.96004 | Hy Bond | Pi-Alkyl |
|  | A:LYS174 | 4.06447 | Hy Bond | Alkyl |
|  | A:TYR223 | 5.27583 | Hy Bond | Pi-Alkyl |
|  | A:ILE224 | 4.59513 | Hy Bond | Amide-Pi Stacked |
|  | A:PRO225 | 5.04014 | Hy Bond | Alkyl |
|  | A:PRO225 | 3.76677 | Hy Bond | Pi-Alkyl |
|  | A:TRP243 | 5.19091 | Hy Bond | Pi-Alkyl |
| 6JBI vs. Chaetoviridin-A | A:ILE251 | 5.39916 | Hy Bond | Alkyl |
|  | A:ARG289 | 2.9139 | H Bond | Conventional H Bond |
|  | A:LYS294 | 3.21822 | H Bond | Conventional H Bond |
|  | A:ARG289 | 4.06166 | Hy Bond | Alkyl |
|  | A:LEU392 | 4.64249 | Hy Bond | Pi-Alkyl |
| 6JBI vs. Camptothecin | A:LEU371 | 3.81587 | Hy Bond | Pi-Sigma |
|  | A:VAL287 | 3.5457 | H Bond | Carbon H Bond |
|  | A:ASN391 | 3.2327 | H Bond | Conventional H Bond |
|  | A:LEU392 | 3.05436 | H Bond | Conventional H Bond |
|  | A:LEU392 | 4.07971 | Hy Bond | Alkyl |
|  | A:VAL393 | 3.37751 | H Bond | Conventional H Bond |
|  | A:VAL393 | 5.331 | Hy Bond | Pi-Alkyl |
|  | A:LYS294 | 2.80268 | H Bond | Conventional H Bond |
|  | A:GLU396 | 4.75471 | Electrostatic | Pi-Anion |
| 6JBI vs. Rocaglaol | A:TYR99 | 2.81774 | H Bond | Conventional H Bond |
|  | A:TYR99 | 4.98159 | Hy Bond | Pi-Alkyl |
|  | A:TRP108 | 4.81824 | Hy Bond | Pi-Alkyl |
|  | A:HIS112 | 4.56601 | Hy Bond | Pi-Alkyl |
|  | A:HIS155 | 4.15648 | Hy Bond | Pi-Alkyl |
|  | A:THR182 | 3.50867 | H Bond | Carbon H Bond |
|  | A:ARG327 | 4.72937 | Electrostatic | Pi-Cation |
|  | A:ASP388 | 3.59608 | H Bond | Carbon H Bond |
|  | A:LEU392 | 5.05223 | Hy Bond | Pi-Alkyl |
| 5E9G vs. Camptothecin | A:LEU434 | 4.95552 | Hy Bond | Pi-Alkyl |
|  | A:TRP440 | 4.5486 | Hy Bond | Pi-Pi T-shaped |
|  | A:ILE466 | 3.35866 | Hy Bond | Pi-Sigma |

H= Hydrogen, Hy= Hydrophobic

**Table S3:** List of studied enzymes and the roles in blast fungus *Magnaporthe oryzae.*

| **Name** | **Pathway** | **Function** | **Reference** |
| --- | --- | --- | --- |
| **Scytalone reductase**  (PDB ID: 1STD) | Melanin biosynthesis pathway. | Appressorium formation. | Kihara et al. 2004  Perpetua et al. 1999 |
| **Trihydroxy**  **naphthalene reductase** (PDB ID: 1YBV) | Melanin biosynthesis pathway. | Appressorium formation. | Jordan et al. 2001 |
| **Trehalose-6-phosphate synthase 1 or Tps1**  (PDB ID: 6JBI) | TPS/TPP pathway | Carbon and nitrogen metabolism during asexual development. | Wilson et al. 2007;  Fernandez and Wilson. 2011; Badaruddin et al. 2013 |
| **Isocitrate lyase**  (PDB ID: 5E9G) | Glyoxylate cycle | Play essential role in pathogen virulence germ tube such as emergence, appressorium development, and cuticle penetration, colonization in host cell and development disease in plant. | Idnurm and Howlett 2002; Wang et al. 2003;  Shin et al. 2007;  Joshi etal. 2020 |
